# Towards a Tensor Product Structure-Grounded Mereology

**DOI:** 10.3390/e28060627

**Published:** 2026-06-02

**Authors:** Matías Pasqualini, Sebastian Fortin

**Affiliations:** 1 CONICET, Instituto de Investigaciones “Dr. Adolfo Prieto”, Universidad Nacional de Rosario, Rosario 2000, Argentina; matiaspasqualini@gmail.com; 2 CONICET, Instituto de Filosofía “Dr. Alejandro Korn”, Universidad de Buenos Aires, Buenos Aires 1406, Argentina

**Keywords:** Hilbert space, mereology, quantum mechanics, tensor product

## Abstract

This paper aims to lay some groundwork for a systematic framework for quantum mereology based on the tensor product structures (TPSs) of Hilbert space. While previous work has suggested that such a TPS-based mereology might conform to classical extensional mereology, we demonstrate that the space of all possible tensor product structures for a given Hilbert space lacks the lattice-theoretic structure characteristic of classical partitions. Specifically, we show that this space admits no canonical meet operation, thus violating the global validity of a natural extension of the weak supplementation principle. These structural features suggest that quantum mereology, even when based on TPSs, should exhibit certain non-extensional behavior: what counts as a “part” is decomposition-relative in a stronger sense, with different decompositions allowed to be mutually incompatible. The resulting picture challenges extensional interpretations of quantum composition and underscores the need for a formally richer, genuinely non-classical mereology.

## 1. Introduction

A longstanding problem in the foundations of quantum mechanics concerns the status of mereology in the quantum domain. Standard mereological frameworks are extensional: strong supplementation guarantees that the identity of a whole is fixed by the identity of its parts. While some authors maintain that such frameworks can be applied to quantum systems, several structural features of quantum theory put this claim under pressure. In particular, the non-Boolean structure of algebras of observables and the distinctive identity conditions associated with indistinguishable quantum systems suggest that quantum composition may fail to be extensional. This casts doubt on the adequacy of extensional mereology for representing quantum composition and leaves open the question of whether a genuinely quantum mereological framework is required. The existing bibliography identifies several core challenges:

(a) The identity conditions of quantum systems.

Systems composed of particles of the same type can only be assigned symmetric (bosonic) or antisymmetric (fermionic) states. As a consequence, distinct quantum systems of the same type are completely indiscernible (i.e., they are only numerically distinct). This feature—quantum indistinguishability—conflicts with extensionality: one may replace a part with another numerically distinct yet indiscernible part without altering the whole [1]. Classical mereology, grounded ultimately in classical logic and the Zermelo–Fraenkel set theory (where identity is purely extensional), is essentially “Leibnizian”: indiscernibility entails identity.

(b) Classical mereology is blind to structure.

Classical mereology does not account for internal relations among parts: two systems with the same parts are identical [1]. It fails to capture compositional structure; it merely imposes minimal constraints to secure extensionality (e.g., strong supplementation). For instance, extensional mereology does not distinguish two molecules of different substances composed of the same atoms arranged differently (e.g., ethanol and dimethyl ether). By contrast, quantum wholes have rich internal structure encoded either by the lattice of Hilbert subspaces (for properties) or by the tensor product factorizations of Hilbert space (for subsystems). A quantum mereology must capture at least one of these.

(c) Quantum holism or non-separability.

As emphasized by Teller [2], Howard [3], Healey [4], Esfeld [5], and others, composite quantum systems can appear in entangled states that are not eigenstates of observables of the parts but are eigenstates of observables of the whole. Quantum wholes can thus possess properties that cannot be decomposed into properties of the parts. Some interpret this as a challenge to extensional mereology; others see it as undermining the very notion of “part” in the quantum domain, since in entangled systems there is no assignment of subsystem states from which the state of the whole can be recovered.

(d) Superpositions of different particle numbers.

Since quantum systems may be prepared in superpositions of states with different particle numbers, and particle number is often taken as a proxy for the number of parts, this seems to threaten any mereology grounded on particle number [6].

We clarify our approach to these issues from the outset. Regarding point (a), we acknowledge that the identity conditions of quantum systems pose a genuine challenge for quantum mereology. Attempts to treat such objects within classical logic or set theory encounter technical and conceptual obstacles, motivating proposals such as Holik and Jorge’s [7] use of quasi-set theory (see also [8,9]), which allows for numerically distinct yet indiscernible objects. However, it is important to note that quantum indistinguishability is not dictated by the Hilbert space structure itself: it derives from the imposition of the symmetrization postulate as a superselection rule motivated by quantum statistics. Since this postulate is not forced by Hilbert space alone, we set issues of identity aside in the present work. Our aim is to show that significant progress can be made by focusing first on structural constraints that quantum mechanics imposes on mereology independently of quantum indistinguishability, namely, the structure of properties (subspaces) and the structure of subsystems (tensor factors). Thus, in this paper, we address challenge (b) and leave (a) for future work.

Addressing (b) requires specifying which Hilbert space structures are relevant to quantum mereology; that is, what should play the role of quantum parts. Two approaches are available:

▪ Taking Hilbert subspaces as parts;
▪ Taking Hilbert tensor factors as parts.

The first option aligns with quantum logic [10], given that subspaces represent quantum propositions. This yields a mereology of quantum properties, relevant for ontologies that treat properties as fundamental [11] or for mereological bundle theory applied to relational quantum mechanics [12]. This is the route taken by Holik and Jorge [7], whose proposal we review in Section 2.1. The second option mirrors standard physical practice, treating subsystems as represented by tensor factors of Hilbert space. This approach is pursued by Calosi and Tarozzi [13], who argue that a mereology grounded on tensor factors corresponds to closed extensional mereology (CEM) or even general extensional mereology (GEM), also known as classical mereology. In this paper, we adopt this second strategy, taking tensor product structures (TPSs) in Hilbert space as the relevant objects a quantum mereology should track, thereby developing a mereology of subsystems. This continues earlier work emphasizing the foundational importance of TPSs [14,15,16,17,18,19]. We return, however, to assess Calosi and Tarozzi’s claim regarding the classical behavior of quantum mereology in Section 4.

Concerning core challenge (c), once TPSs—not subspaces—are taken as the relevant mereological structure, quantum holism becomes less threatening. Although certain global properties (eigenstates of the whole) cannot be factorized into properties (eigenstates) of the parts, the whole (the Hilbert space) is typically factorizable into tensor factors corresponding to subsystems. Of course, this does not make tensor factorizations classical, since the tensor product is not a Cartesian product—it includes linear combinations of product states—so quantum mereology is structurally different from classical mereology, as discussed in Section 4.2. Quantum holism arises when one attempts to align the lattice of properties with a fixed TPS; some states of the whole cannot be factorized relative to a given TPS. But this problem concerns the interaction between properties and a chosen decomposition, not the mereological significance of TPSs themselves.

Finally, regarding (d), one might attempt to build a quantum mereology around the particle number operator. Such an approach could offer a principled way of connecting mereological structure with physically significant observables. However, we set this possibility aside here for reasons of methodological clarity and focus. In our view, the particle number can be interpreted as a global property related to the system’s energy, rather than as directly specifying the number of mereological parts. Moreover, even when the system is in a superposition of different particle number eigenstates, one may choose a definite tensor decomposition of the Hilbert space. From this perspective, the existence of number-superposed states threatens particle number-based mereology but not TPS-based mereology. A fuller assessment of particle number-based approaches is an interesting direction for future work.

These considerations motivate a shift in focus: rather than trying to encode quantum properties or particle numbers into a mereological framework, we propose that the relevant structure for quantum (de)composition is provided directly by the tensor product architecture of Hilbert space. A mereology grounded on tensor factors promises to capture how quantum systems decompose into subsystems while avoiding the conceptual difficulties tied to holism or particle number superpositions.

Therefore, in this paper, we concentrate on the structural constraints that quantum mechanics imposes on mereology and set the basics for the development of a framework grounded on tensor product structures of Hilbert space. We begin in Section 2 by reviewing two existing approaches to quantum mereology: one based on the lattice of Hilbert subspaces and one based on tensor factors. Section 3 surveys the core principles of extensional mereology and introduces the lattice of classical partitions as a benchmark for any theory of (de)composition. Section 4 then articulates some requirements that a TPS-grounded mereology must satisfy, examining the basic correspondence between tensor factors and mereological structure and analyzing the space of tensor product structures available for a given Hilbert space. The aim is to clarify what a quantum mereology based on subsystems should capture, and to assess its prospects in light of both classical constraints and quantum-specific structural features. We provide fresh arguments showing that quantum mereology, even when based on tensor product structures, has non-classical features that extant formal systems of mereology fail to capture.

## 2. Two Different Approaches to Quantum Mereology

The study of quantum mereology—how parts relate to wholes in quantum systems—has developed along two distinct methodological lines. One focuses on the algebraic structure of quantum properties, while the other focuses on the (de)compositional structure of quantum subsystems. These correspond to two different interpretations of what a “part” means in quantum theory: a part as a set of possible properties, or a part as a factor in a tensor product decomposition.

### 2.1. Approach Grounded on Hilbert Subspaces

Holik and Jorge [7] take the logical and algebraic structure of propositions about quantum systems as their starting point. In classical mechanics, the state of a composite system is simply the Cartesian product of the states of its subsystems, and the lattice of propositions is distributive. This allows a straightforward mapping between the propositions of the whole and those of its parts via canonical projections. In quantum mechanics, however, the lattice of propositions—represented by closed subspaces or projection operators—is non-distributive and orthomodular, failing to provide a satisfactory part–whole relation for composite systems. When a composite system is in an entangled pure state, its subsystems are described by improper mixtures, which do not correspond to any element of the subsystem’s projector lattice.

To overcome this limitation, Holik and Jorge propose extending the notion of proposition associated with a quantum system from actual to probabilistic assertions. This way, they extend the lattice of quantum propositions to include convex subsets of the space of density operators. The central conceptual move is to take convex sets of states as the primary mereological relata. In doing so, they effectively treat quantum properties—or more precisely, families of states sharing a probabilistic attribute—as the “parts” of a system.

Let C be the set of all density operators. Probabilistic assertions are defined as follows:

(1) $$C_{E}\left(p\right)\;=\;\left\{\rho \in C|\mathrm{tr}\left(\rho E\right)=p\right\}$$

where *E* is a quantum property represented by a projector operator and C are convex subsets of C. These probabilistic assertions form a lattice $L_{C}$ under suitably defined meet, join, negation, and implication operations. This structure, while not an ortholattice (double negation does not hold), contains the original lattice of projections as a sublattice and accommodates mixed states as legitimate propositions.

Using these extended lattices, Holik and Jorge define canonical maps between the lattice of the composite and those of the subsystems $L_{C_{1}},\;L_{C_{2}},\;L_{C_{1}}\times L_{C_{2}},\;L_{C}$. These maps formally capture the non-reducibility of the properties of the whole to the properties of its parts. In particular, they highlight the non-commutativity of the operations of “going from the whole to the parts” and “going from the parts to the whole,” expressed by the relation

(2) Λ∘τ≠τ∘Λ

where Λ is the map from $L_{C_{1}}\times L_{C_{2}}$ to $L_{C}$ and τ is the map from $L_{C}$ to $L_{C_{1}}\times L_{C_{2}}$. This inequality, indicating the non-commutativity of the mappings between the properties of the whole and the properties of the parts, encapsulates the non-extensional character of mereological relations between quantum properties: the information in the whole is not simply the sum of the information in the parts.

This approach provides a rigorous extension of quantum logic and elegantly encodes the holistic features of quantum systems, formally expressing the failure of reducibility in a lattice-theoretic language. In this framework, the part–whole relation becomes a relation between probabilistic properties of the whole and those of the subsystems. It provides the mathematical underpinnings for a mereology of quantum properties, whose non-extensionality flows naturally from the fact that it is an extension of quantum logic. However, one may also pursue a subsystem-based quantum mereology that takes tensor factors—or more generally, subalgebras of observables—as its primitive notion of “part”. Such an approach would stay closer to the operational and ontological commitments of physical practice, where the following occurs:

▪ Composition is modeled by tensor products: Putting systems together corresponds to taking the tensor product of their Hilbert spaces.
▪ Subsystems retain algebraic identity: Even when their state is mixed or undefined, a subsystem is associated with a well-defined algebra of local operators.

This is the stance toward quantum mereology clearly taken first by Calosi and Tarozzi [13]. In their article, Calosi and Tarozzi attempt to determine which mereological theories are a model of quantum systems. They argue that quantum systems satisfy the axioms of extensional mereology (EM) and possibly stronger closure mereologies, such as closure extensional mereology (CEM) or even general extensional mereology (GEM).

### 2.2. Approach Grounded on Hilbert Tensor Factors

Before moving on, let us briefly review the mathematics of tensor products in Hilbert space [20]. Given two quantum systems $S_{1}$ and $S_{2}$ with associated Hilbert spaces $H_{1}$ and $H_{2}$, the state space for the joint system S is not their Cartesian product, but a more structured object known as the tensor product space, denoted $H≅H_{1}⊗H_{2}$. This space is formally built from a bilinear pairing $⊗:H_{1}\times H_{2}\to H$ that satisfies two fundamental axioms:

▪ Generative Property: The set of all simple tensors $\left\{|\psi_{1}\rangle ⊗|\psi_{2}\rangle \right\}$ spans the entire space H. This means any state of the composite system can be expressed as a linear superposition of such product states.

▪ Metric Preservation: The inner product on the composite space is defined in a factorized manner:

(3) $${\langle \psi_{1}⊗\psi_{2}|\varphi_{1}⊗\varphi_{2}\rangle }_{H}={\langle \psi_{1}|\varphi_{1}\rangle }_{H_{1}}\cdot {\langle \psi_{2}|\varphi_{2}\rangle }_{H_{2}}$$

This axiom ensures that the geometry (and thus the probabilistic interpretation) of the individual spaces is faithfully embedded into the composite one. The inner product extends by linearity from the spanning set to all of H, completing its definition as a Hilbert space.

A critical consequence of this construction is that $H_{1}⊗H_{2}$ is significantly larger than the mere set of product states. Its vectors are generally linear combinations thereof, leading to the aforementioned entangled states and quantum holism.

Operators representing physical observables inherit the tensor product structure. If $O_{1}$ acts on $H_{1}$ and $O_{2}$ acts on $H_{2}$, their joint action on H is defined by the linear operator $O_{1}⊗O_{2}$. For the description of a subsystem within the composite, we require operators that act non-trivially on only one factor. These are of the form $O_{1}⊗I_{2}$ and $O_{2}⊗I_{1}$, where I denotes the identity operator. The algebra generated by all operators of the form $O_{1}⊗I_{2}$ constitutes the observable algebra for subsystem $S_{1}$ as viewed from the total system. This defines a subalgebra of the full algebra of bounded operators on H, providing a precise algebraic characterization of the subsystem.

A fundamental and often underemphasized aspect of composition in quantum theory is that a given Hilbert space H generally admits multiple, physically distinct tensor product factorizations. This means that what constitutes a “subsystem” is not an absolute property of the Hilbert space itself, but rather a choice of decomposition tied to physical degrees of freedom. Formally, if $dim\left(H\right)=N$ is a composite integer $\left(N=n_{1}\times n_{2}\right)$, there exists multiple ways to express H as a tensor product:

(4) $$H≅{\mathbb{C}}^{n_{1}}⊗{\mathbb{C}}^{n_{2}}$$

The isomorphism is not canonical. Different choices of basis—or, more fundamentally, different identifications of what constitutes the “local” degrees of freedom—lead to different tensor product structures (TPSs). It is important to note that, in formulas where the tensor product appears, it is convenient not to employ the equality or identity symbol (=), but rather the weaker symbol for equivalence (≅). Different TPSs are not identical but equivalent; each of them points to a different internal structure not captured by the others. This will become clearer when we consider the non-extensionality of the space generated by all TPSs of a single whole.

Given an orthonormal basis ${\left\{|k\rangle \right\}}_{k=1}^{N}$ for H, one may define a TPS by partitioning the basis into two sets and mapping them to product bases. For example, a bipartite factorization can be established via a bijection:

(5) $$|k\rangle ↔{|i\rangle }_{1}⊗{|j\rangle }_{2},with\;k↔\left(i,j\right)$$

where the specific pairing $\left(i,j\right)$ is arbitrary. Each pairing defines a distinct algebraic subdivision of the total operator algebra into commuting subalgebras associated with the subsystems $S_{1}$ and $S_{2}$.

While some authors maintain that a privileged tensor product structure can be objectively selected by adopting one criterion or another (e.g., by minimizing entanglement, Carroll and Singh [21]), the multiplicity of possible tensor product structures (TPSs) a quantum system generally admits has prompted a relatively recent and growing line of research in the foundations of quantum mechanics, often termed the TPS approach. This perspective highlights the fact that many core quantum-theoretical notions are decomposition-relative:

▪ Entanglement, as Earman [17] notes, is not an intrinsic property of a quantum state, but depends on the selected TPS. A state entangled with respect to one factorization may be separable with respect to another.
▪ Particle statistics may also be TPS-dependent. As argued by Pasqualini and Fortin [18], a given global state can be symmetric under particle permutation in one TPS and antisymmetric in another, challenging the idea that particle labels have an absolute physical meaning independent of the chosen algebraic decomposition.
▪ Most fundamentally, the very notion of separability and the identity of subsystems becomes relative. What counts as a “part” or a “subsystem” is not fixed by the Hilbert space alone but is a consequence of the chosen TPS [14,15,16].

This relativity fundamentally challenges the assumption that physical systems generally possess a classically defined part–whole structure, suggesting that any quantum mereology grounded in the TPS formalism must explicitly incorporate and reflect the fact that decomposition—and therefore what constitutes a “part”—is itself TPS-relative.

By taking tensor factors in Hilbert space as quantum parts, Calosi and Tarozzi [13] develop a mereological framework broadly aligned with the tensor product structure (TPS) approach. Their analysis is not explicitly concerned, however, with the multiplicity of possible decompositions. Instead, they proceed by investigating whether quantum systems—interpreted via a given TPS—satisfy the core axioms and supplementation principles of classical extensional mereology.

▪ Reflexivity: Whether a quantum system is part of itself is seen as a matter of convention. Reflexivity can be preserved by distinguishing proper parthood (irreflexive) from parthood (reflexive). Thus, reflexivity is considered safe.
▪ Transitivity: They provide a Hilbert space argument for transitivity. If $S_{1}$ is part of $S_{2}$ and $S_{2}$ is part of $S_{3}$, then the Hilbert space of $S_{3}$ is constructed via tensor products involving $H_{1}$. Using the Schmidt decomposition, they show that every state of $S_{3}$ depends on contributions from $S_{1}$, implying that $S_{1}$ is part of $S_{3}$.

▪ Weak Supplementation Principle (WSP): WSP states that a composite entity cannot have exactly one proper part. They argue that if a quantum system S had only one proper part $S_{1}$, then S would have to be described by a Hilbert space $H=H_{1}⊗H_{n}$ for some $H_{n}$. Since no such $H_{n}$ exists (by hypothesis), S would lack a quantum-mechanical description—an unacceptable outcome. Thus, quantum systems do not violate WSP.

▪ Strong Supplementation Principle (SSP): SSP states that if *y* is not part of *x*, then there exists some part of *y* disjoint from *x*. Calosi and Tarozzi argue that in composite systems S, composed of $S_{1},S_{2}$ via a tensor product, the subsystems $S_{1}$ and $S_{2}$ are disjoint because the reduced state of $S_{1}$ (obtained via partial trace) contains no contributions from $H_{2}$. This holds even if the subsystems share a common part $S_{3}$; in that case, further decomposition and application of WSP ensure disjointness. Hence, SSP is satisfied.

From these arguments, they conclude that quantum systems are models of extensional mereology (EM). Calosi and Tarozzi also consider composition principles:

▪ Binary Sum Principle (BSP): Given two quantum systems $S_{1}$ and $S_{2}$ that underlap (i.e., are parts of some whole), their mereological sum is naturally identified with the composite system whose states are constructed from tensor products of states in $H_{1}$ and $H_{2}$. In their words, “every state of S is built up from all the resources of $H_{1}$, $H_{2}$ and nothing else”. This composite system overlaps exactly those entities that overlap $S_{1}$ or $S_{2}$, satisfying BSP. Thus, quantum systems also model closure extensional mereology (CEM).

▪ Unrestricted Composition (UC): While they do not definitively endorse UC, their framework suggests that any collection of quantum systems has a mereological sum describable within the tensor product structure of Hilbert spaces. This would align quantum systems with general extensional mereology (GEM).

By arguing that quantum systems satisfy extensional mereology, Calosi and Tarozzi push back against the view that quantum mechanics requires a radical revision of classical mereology. Instead, they suggest that at the most fundamental level, parthood behaves extensionally: a system’s identity is completely determined by its parts. This claim contests the typical discourse about the failure of extensionality due to quantum holism and quantum indistinguishability. Moreover, by equating mereological composition with the tensor product operation in Hilbert space, they offer a concrete physical procedure for understanding what it means for parts to form a whole: a mathematically well-defined construction that preserves the information and structure of the subsystems.

We agree with Calosi and Tarozzi that quantum mereology should be grounded in tensor product structures, as this reflects actual physical practice—subsystems are identified with tensor factors, and composition is modeled via tensor products. However, we depart from their conclusion that quantum mechanics provides a model of extensional mereology (CEM). Our contention is that the space of possible tensor factorizations of a given Hilbert space is far richer and more structurally complex than the lattice of partitions in classical mereology. In particular, as we will argue in Section 4.2, this space lacks a global lattice structure: there is no unique, canonical way to reconcile the different decompositions of a quantum whole, since they can be incompatible or non-orthogonal in a way that violates extensionality. This indicates that quantum mereology, even when based on tensor factors, should be non-extensional and richer than classical mereological theories. Calosi and Tarozzi’s extensional conclusion overlooks this structural richness by implicitly privileging a single factorization scheme, thereby missing the multiplicity of possible decompositions that quantum mechanics permits.

## 3. Reviewing Extensional Mereologies

### 3.1. Core and (De)Composition Principles

In mereology, one can distinguish between systems that assume only basic postulates (the so-called fundamental or ground mereology) and others that are committed to more substantive principles, such as the weak supplementation principle and the strong supplementation principle. These give rise, respectively, to what is called minimal mereology (MM, for minimum mereology) and extensional mereology (EM, for extensional mereology). If one adds to EM the so-called principle of unrestricted composition, one obtains what is known as general extensional mereology (GEM, for general extensional mereology), also known as standard or classical mereology, a theory with great expressive power, almost as strong as standard set theory [22].

The basic mereological notion is the notion of part. The fundamental mereological relation is the parthood relation, which mediates between a part and the whole it belongs to. Let *P* be a binary predicate representing the parthood relation. Thus, for example, *Pxy* is read as “*x* is part of *y*.” Since *P* is intended to constitute a partial order, the relation *P* is characterized by Varzi [22]:

▪ Reflexivity (R): ∀xPxx

▪ Transitivity (T): $\forall x\forall y\forall z\left(Pxy\wedge Pyz\to Pxz\right)$

▪ Antisymmetry (AS): $\forall x\forall y\left(Pxy\wedge Pyx\to x=y\right)$

The theory comprising only these three axioms is termed ground mereology (M). Based on these three mereological postulates, one can proceed to define some additional mereological notions:

▪ Proper Parthood $PPxy\equiv Pxy\wedge \neg \left(x=y\right)$

From the conjunction of (R), (T), and (AS), it follows that the parthood relation (*P*) constitutes a partial order. The proper parthood relation (*PP*), due to its irreflexivity, constitutes a strict partial order [21]. Thus, *PP* allows one to define a strict partial order over the domain of the parts of an object, for example, *u*. Let $\left\{x,y\right\}$ be any parts of *u*. If it is the case that PPxy, then $\left\{x,y\right\}$ is an ordered pair. Conversely, if it is the case that PPyx, then $\left\{y,x\right\}$ is an ordered pair.

On the other hand, it is possible to define the following relations:

▪ Overlap $Oxy\equiv \exists z\left(Pzx\wedge Pzy\right)$

Two entities overlap if they share a common part.

▪ Disjointness Dxy≡¬Oxy

Two entities are disjoint if they do not share parts.

▪ Underlap $Uxy\equiv \exists z\left(Pxz\wedge Pyz\right)$

Two entities underlap if they are both parts of some common entity.

▪ Atom Ax≡¬∃yPPyx

An atom has no proper parts.

Ground mereology can be strengthened by adding principles governing how a whole relates to its parts. A fundamental, weak principle is as follows:

▪ Weak Supplementation Principle (WSP):

$$\forall x\forall y\left(PPxy\to \exists z\left(Pzy\wedge Dzx\right)\right)$$

If an object has a proper part, it must have another part disjoint from the first. Adding WSP to R and T yields minimal mereology (MM).

A stronger and more philosophically significant principle is as follows:

▪ Strong Supplementation Principle (SSP):

$$\forall x\forall y\left(\neg Pyx\to \exists z\left(Pzy\wedge Dzx\right)\right)$$

If *y* is not a part of *x*, then *y* has some part wholly disjoint from *x*. SSP entails WSP (but not vice versa) and, when added to the lexical axioms, results in extensional mereology (EM). The “extensional” label stems from the theorem that, under SSP, composite objects with identical proper parts are themselves identical.

While decomposition principles move from whole to parts, composition principles move from parts to whole, guaranteeing the existence of sums or fusions.

▪ Binary Sum Principle (BSP):

$$\forall x\forall y\left(Uxy\to \exists z\forall w\left(Owz↔\left(Owx\vee Owy\right)\right)\right)$$

This axiom asserts that any two underlapping entities have a unique mereological sum z=x+y. Adding BSP to MM gives closure minimal mereology (CMM) and adding it to EM gives closure extensional mereology (CEM).

The most comprehensive and debated composition principle is:

▪ Unrestricted Composition (UC):

$$\exists w\left(\varphi w\right)\to \exists z\forall w\left(Ozw↔\exists v\left(\varphi v\wedge Owv\right)\right)$$

For any non-empty domain of objects satisfying φ (where φ is an open formula), there exists an entity *z* that is the mereological sum of all φ-ers. Adding UC to EM produces general extensional mereology (GEM), also known as classical or standard mereology.

A critical metaphysical question is which ontological domains, including the domain of physical systems, instantiate which mereological theory. The answer is not straightforward, as the logical operations implicit in mereological definitions (conjunction, disjunction, existential quantification) may behave differently in different physical theories. In classical mechanics, the algebra of properties is Boolean, aligning neatly with classical logic. In quantum mechanics, however, the structure of properties is non-Boolean, leading to the various formalisms of quantum logic. This has direct implications for interpreting mereological axioms in a quantum context, especially if one is willing to take Hilbert subspaces as quantum parts. For instance, the disjunction in the definition of a sum must be treated with care: the quantum proposition “*w* overlaps with *x* or *y*” is not equivalent to “*w* overlaps with *x* or *w* overlaps with *y*” if the disjunction is interpreted as a quantum join (span). Consequently, the validity of mereological principles like WSP, SSP, BSP, or UC cannot be assumed a priori for quantum systems but must be critically examined within the (de)compositional structure of quantum theory.

### 3.2. Extended Framework for Mereological Partitions and Their Lattice Structure

While the standard apparatus of classical mereology provides tools for discussing individual parts and their sums, the systematic study of ways of dividing a whole into parts—mereological partitions—has received less attention. In this subsection, we suggest that under appropriate conditions, the collection of all mereological partitions of a given whole forms a lattice structure, analogous to the partition lattices familiar from set theory. We extend closed extensional mereology (CEM) by defining some additional notions.

▪ Mereological Partition: a set $\alpha =\left\{a_{1},a_{2},\ldots ,a_{n}\right\}$ of objects is a mereological partition of an object *u* if the elements of α are parts of *u*, are mereologically disjoint, and every part *w* of *u* is part of one of the elements of α or of the mereological sum of some of the elements of α.

That is, α is a mereological partition of *u* if

(1) Parthood: The elements of α are parts of *u*:
  (6) $$\forall x\left(x\in \alpha \to Pxu\right)$$

(2) Disjointness: Distinct elements of α are pairwise mereologically disjoint:

(7) $$\forall x\forall y\left(x\in \alpha \wedge y\in \alpha \wedge x\neq y\to Dxy\right)$$

(3) Covering: Every part *w* of *u* is part of the mereological sum (*S*) of some subset β of α.

(8) $$\forall w\left(Pwu\to \exists \beta \subseteq \alpha \left(P\left(w,S\left(\beta \right)\right)\right)\right)$$

where $S\left(\beta \right)$ denotes the mereological sum of the elements of β. The sum condition ensures the partition covers the whole. Taken together, the parts compose the entire object. The disjointness condition ensures they do not overlap.

This definition of mereological partition bears a relation to the notation used in some works in quantum physics, according to which, if $S^{1},S^{2}\ldots S^{N}$ are subsystems of U, then $S^{1}\cup S^{2}\cup \ldots S^{N}=U$, such that $S^{i}$ resemble the mereological parts of U and ∪ resembles the mereological sum. Note that $S^{1}\cup S^{2}\cup \ldots S^{N}=U$ satisfies conditions (1), (2), and (3) required by the definition of mereological partition:

(i) If $S^{1}\cup S^{2}\cup \ldots S^{N}=U$, then $S^{1}\subseteq U$, $S^{2}\subseteq U$… $S^{N}\subseteq U$, satisfying (1)

(ii) It is assumed that $S^{i}\cap S^{j}=\emptyset$, satisfying (2)

(iii) If W⊆U and W≠∅, then $W\subseteq \left(S^{i}\cup S^{j}\cup \ldots S^{n}\right)$, satisfying (3).

The set of all mereological partitions of *u* is denoted $\Pi \left(u\right)$. Earlier reference was made to the fact that the parthood relation (*P*) induces a partial order on the domain of the parts of an object *u*. Before proceeding, it is useful to define a notion of partial order on the domain of the mereological partitions of the object *u*, the set $\Pi \left(u\right)$. Different partitions of the same whole can be compared according to their granularity. A partition ε is finer than a partition ϕ if each block of ε is contained within some block of ϕ.

▪ Refinement Order: For partitions $\varepsilon ,\phi \in \Pi \left(u\right)$ we say ε refines ϕ, written $\varepsilon \underline{≺}\phi$, if for every part x∈ε there exists a part y∈ϕ such that Pxy:

(9) $$\varepsilon \underline{≺}\phi \equiv \forall x\exists y\left(x\in \varepsilon \wedge y\in \phi \to Pxy\right)$$

In other words, ε is at least as fine-grained as ϕ. This relation is clearly reflexive: every block of ε is part of itself. It is also transitive: if each block of ε is part of some block of ϕ, and each block of ϕ is part of some block of ψ, then each block of ε is part of some block of ψ.

To ensure antisymmetry—that $\varepsilon \underline{≺}\phi$ and $\phi \underline{≺}\varepsilon$ together imply ε=ϕ—we need the fact that distinct blocks within a partition are disjoint. If two partitions refine each other, their blocks must coincide pairwise; else, we would have overlapping blocks. Thus $\underline{≺}$ is a partial order on $\Pi \left(u\right)$.

Every partially ordered set of partitions grounded on classical mereology naturally exhibits extremal elements:

▪ The coarsest possible partition is the one that does not divide the whole at all: just $\left\{u\right\}$, the whole itself is treated as a single block. Clearly, for any partition ε, each block of ε is part of *u*, so $\varepsilon \underline{≺}\left\{u\right\}$. This is the top element of our ordering.

▪ The finest possible partition requires that the whole be composed of atoms—parts with no proper parts. If we assume atomicity (every object is composed of atoms), then the partition consisting of all atoms of *u* is the finest possible. Each atom is a block, and any block of any other partition must contain whole atoms. Thus, the atomic partition refines every other partition. This is the bottom element. So $\Pi \left(u\right)$ is bounded: it has a greatest element $\left\{u\right\}$ and a least element $\chi_{u}$ (the atomic partition).

Let us recall that a lattice is a partially ordered structure in which every pair of elements has a unique least upper bound (join) and greatest lower bound (meet). Hence, the lattice structure of $\Pi \left(u\right)$ emerges through the definition of binary meet and join operations:

▪ Construct the meet ε∧ϕ by taking, for each pair of blocks x∈ε and y∈ϕ that overlap, their mereological product x×y is the largest common part of *x* and *y*. In classical mereology, this product exists whenever two objects overlap. The collection of all such non-empty products forms a partition: each product is part of *u* (since it is part of both *x* and *y*), and different products are disjoint because they derive from distinct pairs of blocks from the original partitions, which are themselves disjoint, and together they sum to *u* (every atom of *u* belongs to exactly one such product). Moreover, this partition is the greatest lower bound of ε and ϕ: it refines both, and any partition refining both also refines it.

▪ To construct the join ε∨ϕ, consider the atoms of *u*. Two atoms are considered connected if they belong to the same block in ε or the same block in ϕ. Take the transitive closure of this connection relation to get the equivalence classes of atoms. Each equivalence class is fused into a single block—the mereological sum of all atoms in that class. These fused blocks form a partition: they are parts of *u*, disjoint (atoms from different classes are not connected), and together they sum to *u*. This partition is the least upper bound of ε and ϕ: both refine it, and it refines any partition that both ε and ϕ refine.

With meet and join defined, the structure $L\left(u\right)=\left(\Pi \left(u\right),\underline{≺},\wedge ,\vee ,\chi_{u},\left\{u\right\}\right)$ is a bounded lattice. The proof would require checking that the lattice axioms (idempotency, commutativity, associativity, absorption) are satisfied by the construction of meet and join. When *u* has finitely many atoms, this lattice is actually isomorphic to the partition lattice of the set of atoms. Each mereological partition corresponds to a set partition of the atoms, and the refinement order matches perfectly.

We finally define mereological hierarchies as maximal chains within this lattice structure:

▪ Mereological Hierarchy: A mereological hierarchy of *u* is a subset $K\subseteq \Pi \left(u\right)$. That is:

(1) Linearly ordered by $\underline{≺}$;

(2) Complete: if $\lambda \subseteq \Pi \left(u\right)$ is comparable in terms of refinement with an element of K, then λ∈K.

## 4. Requirements for a TPS-Grounded Quantum Mereology

### 4.1. Correspondences Between TPSs and the Extended Mereological Framework

Now that we have introduced this extended framework showing that the set of partitions of a given whole has a lattice structure if built upon classical mereology, we turn to assess how this should behave under a TPS-grounded mereology. First, we must specify some correspondences between TPSs and the above extended mereological notions. It is known that a Hilbert space representing a quantum system generally admits a plurality of different tensor product structures. The idea behind a TPS-grounded mereology is that these TPSs can be mapped onto the different mereological partitions admitted by a total object. In this way, we can bring mereological notions closer to the language and working methods used in scientific practice. For this, one has to take the relation that holds between the tensor factors in TPSs and the tensor product Hilbert space as analogous to the mereological parthood relation (*P*) that holds between parts and wholes. The nature of the tensor product guarantees that the tensor factors obtained in each TPS satisfy parthood, disjointness, and covering, which were imposed as the necessary and sufficient conditions for a set of objects to count as a mereological partition of a certain total object (see Equations (6)–(8)).

The notation mentioned above, in which the relation between the subsystems and the total system is expressed as  $S^{1}\cup S^{2}\cup \ldots S^{N}=U$, satisfying conditions for it to be qualified as a mereological partition if certain assumptions are made, is a simplified way of saying the following: there is a collection of quantum systems $S^{i}$, each one represented by the Hilbert space $H_{i}$; when all these systems are considered jointly, they form the total system U, which is represented by the tensor product Hilbert space $H_{U}≅H_{1}⊗H_{2}⊗\ldots H_{N}=\overset{N}{\underset{i=1}{⊗}}H_{i}$. Thus, the mereological whole *u* corresponds to the tensor product Hilbert space $H_{U}$; each mereological part *x* corresponds to a Hilbert tensor factor $H_{i}$; and the mereological partition α is associated with a TPS-α that corresponds to the decomposition  $H_{1}⊗H_{2}⊗\ldots H_{N}$, so that the following can occur:

(i) If in Equation (6) the variable *x* is replaced by a tensor factor $H_{i}$ that results from TPS-α, one finds that these tensor factors satisfy parthood as follows:

(10) $$\forall H_{i}\left(H_{i}\in \alpha \to H_{i}\subseteq H_{U}\right)$$

This is because any tensor factor of a total space will be included in the total space. One may say that the mereological notion of part captures the quantum-mechanical notion of subsystem.

(ii) If in Equation (7) the variables *x* and *y* are replaced by tensor factors $H_{i}$ and $H_{j}$ that result from TPS-α, one finds that these tensor factors satisfy disjointness:

(11) $$\forall H_{i}\forall H_{j}\left(H_{i}\in \alpha \wedge H_{j}\in \alpha \to H_{i}\cap H_{j}=\emptyset \right)$$

since these tensor factors do not, in turn, share any subspace. In physical terms, the subsystems of a given total system must not share degrees of freedom.

At this point, it is important to clarify that the standard use in quantum mechanics usually presupposes that when a tensor product of this kind is written, the factors represent subsystems with independent degrees of freedom. This is natural when composing systems such as the hydrogen atom, where the Hilbert space representing the electron refers to degrees of freedom independent from that representing the nucleus. However, this is not a feature imposed by the tensor product itself, since there is no restriction regarding the possibility of decomposing a Hilbert space into a product of two non-disjoint Hilbert spaces. It is a scientific practice that imposes this way of using the tensor product. Nevertheless, there are cases, such as that of indistinguishable particles, where, due to the symmetrization of the wave function, the degrees of freedom become mixed and, as previously mentioned, will be studied in future work.

(iii) If in Equation (8) the variable *w* is replaced by any tensor factor $H_{w}$ of $H_{U}$ that does not necessarily correspond to a tensor factor in TPS-α, and β is replaced by a tensor factor $H_{\beta }$ of $H_{U}$, which is itself a tensor product of some tensor factors in TPS-α, one finds that covering is satisfied:

(12) $$\forall H_{w}\left(H_{w}\subseteq H_{U}\to \exists H_{\beta }\left(H_{w}\subseteq H_{\beta }\right)\right)$$

since the elements of the tensor factor that takes the place of *w* necessarily appear as tensor factors in the elements of the tensor product generated by some tensor factors in TPS-α. This stems from the fact that all tensor factors in TPS-α generate $H_{U}$. Please note that the structure of Equation (12) closely mimics that of Equation (8), with the mereological sum $S\left(\beta \right)$ of elements in the subset β⊆α in Equation (8) being replaced by the tensor product $H_{\beta }$ of some tensor factors in TPS-α.

Having established, by (i), (ii), and (iii), the correspondence between tensor product structures and the notion of mereological partition, we now assess whether a set of TPSs can satisfy the notion of mereological hierarchy. Note that a tensor factor of a total space whose dimension is not a prime number can, by the same tensor product operation, be further factorized into a number of tensor factors. This makes it reasonable to suppose that, given a total system, there exist different TPSs between which relations analogous to parthood (*P*) may hold among their respective tensor factors. This allows the notion of refinement between mereological partitions, defined earlier (see Equation (9)), to be applied to sets of TPSs. In fact, if in Equation (9) the variables *x* and *y* are replaced by tensor factors $H_{i}$ and $H_{j}$, the former belonging to TPS-ε and the latter to TPS-ϕ, one finds that two TPSs of the same total system can be related by the notion of refinement order $\underline{≺}$ defined below:

(13) $$\varepsilon \underline{≺}\phi \equiv \forall H_{i}\exists H_{j}\left(H_{i}\in \varepsilon \wedge H_{j}\in \phi \to H_{i}\subseteq H_{j}\right)$$

This means that, when two TPSs are related by refinement, every tensor factor in the finer partition is a tensor factor of some tensor factor in the coarser partition. As noted above, as long as the dimension of the factors in a given TPS is not prime, one can further factorize them to obtain finer-grained TPSs. Therefore, it is possible that, given a total system, some of its TPSs constitute sets that satisfy conditions (1) and (2) specified for mereological hierarchies.

Up to this point, solely considering sets of mereological hierarchies comprising only mutually algebraically compatible partitions (families of compatible partitions from now on), it appears that a tensor product structure-grounded mereology should not only satisfy the axioms of closed extensional mereology (CEM), thus constituting a model of such a mereological theory—in line with the proposal of Calosi and Tarozzi—but also the extended framework of CEM presented in this work, in the sense that all mereological partitions of the same whole could be embedded into a single lattice-theoretic structure. We now ask: is the full structure of TPSs in a Hilbert space (the set of all quantum mereological partitions, including mutually incompatible ones) really isomorphic to the lattice of classical partitions? This question will be addressed in the two following and final subsections.

### 4.2. The Space of Tensor Product Structures

Having established the correspondence between tensor product structures (TPSs) and mereological partitions, as well as the applicability of a refinement order to them, we now examine the space of all possible TPSs for a given Hilbert space H representing a quantum system. In Section 3.2, we showed that, given the axioms of closed extensional mereology, the set of mereological partitions $\Pi \left(u\right)$ of a whole *u* forms a bounded lattice $L\left(u\right)$ under the refinement order $\underline{≺}$. If quantum mechanics—once TPSs are taken to be the relevant mereological structure—were genuinely a model of closed extensional mereology, one would expect an analogous result to hold for the set of all tensor product structures of a given Hilbert space. In this subsection, we show that this expectation fails: the space of TPSs does not carry a global lattice structure. We then argue that this failure signals a genuinely non-classical feature of TPS-grounded quantum mereology.

Let H be a Hilbert space with dimH=N<∞, where N is not prime. Our focus here is on finite-dimensional Hilbert spaces. The argument could be extended to infinite-dimensional, separable Hilbert spaces, but that involves subtleties that we cannot address here and are therefore left for future work. Recall that a tensor product structure (TPS) on H is an isomorphism.

(14) $$H≅\overset{k}{\underset{i=1}{⊗}}H_{i}$$

where k≥2 and $\prod_{i}dimH_{i}=N$. As argued in Section 4.1, each TPS naturally corresponds to a mereological partition of the whole system U, whose blocks are the subsystems represented by the tensor factors $H_{i}$. Although all TPSs of a given Hilbert space are mathematically equivalent—that is, the tensor product of any such factorization yields the same product space—they are regarded as distinct decompositions in the mereological sense, giving rise to the space $T\left(H\right)$ of quantum partitions.

Within $T\left(H\right)$, two TPSs $\tau_{1}$ and $\tau_{2}$ belong to different families of compatible partitions if there is no unitary operator that maps each tensor factor of $\tau_{1}$ onto a tensor factor of $\tau_{2}$—i.e., if they correspond to different algebraic decompositions of the total operator algebra into commuting subalgebras. In that case, $\tau_{1}$ and $\tau_{2}$ cannot be ordered by the refinement relation defined above. However, if $\tau_{1}$ and $\tau_{2}$ belong to the same family of algebraically compatible partitions, the notion of refinement $\underline{≺}$, defined in Equation (13), constitutes an applicable partial order. If every factor in $\tau_{1}$ can be obtained by factorizing some factor in $\tau_{2}$, then $\tau_{1}$ is said to be at least as fine-grained as $\tau_{2}$, meaning that they belong to the same mereological hierarchy.

This refinement relation is clearly reflexive: every tensor factor in $\tau_{1}$ is a tensor factor of itself. It is also transitive: if each tensor factor in $\tau_{1}$ is a tensor factor of some tensor factor in $\tau_{2}$, and each tensor factor in $\tau_{2}$ is a tensor factor of some tensor factor in $\tau_{3}$, then each tensor factor in $\tau_{1}$ is a tensor factor of some tensor factor in $\tau_{3}$. To ensure antisymmetry—that $\tau_{1}\underline{≺}\tau_{2}$ and $\tau_{2}\underline{≺}\tau_{1}$ together imply $\tau_{1}=\tau_{2}$—we need the fact that distinct tensor factors within a partition are disjoint, as already shown in Equation (11). If two TPSs refine each other, their tensor factors must coincide pairwise; else, we would have tensor factors sharing a subspace. Thus $\underline{≺}$ constitutes a partial order on $T\left(H\right)$, mirroring the refinement order on classical partitions.

The crucial question is whether $T\left(H\right)$, ordered by refinement, forms a lattice. That is, given two TPSs $\tau_{1}$ and $\tau_{2}$, does there always exist (i) a greatest lower bound (a meet TPS refining both) and (ii) a least upper bound (a join TPS coarser than both)? Let us first address the first question by means of an example.

Let us consider three qubits *A*, *B,* and *C*, represented in Hilbert space $H_{A}$, $H_{B}$ and $H_{C}$, respectively. Together, they form the total system *U* represented by $H≅H_{A}⊗H_{B}⊗H_{C}≅{\mathbb{C}}^{2}⊗{\mathbb{C}}^{2}⊗{\mathbb{C}}^{2}≅{\mathbb{C}}^{8}$. Then, this space admits, among others, the following two bipartite TPSs:

(15) $$\begin{array}{l} \alpha :H≅\left(H_{A}⊗H_{B}\right)⊗H_{C}≅{\mathbb{C}}^{4}⊗{\mathbb{C}}^{2}\\ \beta :H≅H_{A}⊗\left(H_{B}⊗H_{C}\right)≅{\mathbb{C}}^{2}⊗{\mathbb{C}}^{4} \end{array}$$

Note that α and β group the three qubits differently. Neither is maximally fine: each can be further factorized to the tripartite TPS $H_{A}⊗H_{B}⊗H_{C}$. Hence, both α and β lie strictly above finer decompositions in the refinement order, having that tripartite TPS as their meet: $\alpha \wedge \beta =H_{A}⊗H_{B}⊗H_{C}$.

Now, a change in coordinates is performed on β that mixes the degrees of freedom of *B* and *C*. Based on that change in coordinates, a new TPS $\beta^{\prime }$ is defined, in which the four-dimensional factor in β, namely $H_{B}⊗H_{C}$, is replaced by a new factor $H_{D}⊗H_{E}$. In this way, two new subsystems, *D* and *E*, represented by $H_{D}$ and $H_{E}$, respectively, are defined. Together with *A*, they constitute the same total system represented by $H≅H_{A}⊗\left(H_{D}⊗H_{E}\right)≅{\mathbb{C}}^{2}⊗{\mathbb{C}}^{4}≅{\mathbb{C}}^{8}$.

Note that the two decompositions β and $\beta^{\prime }$ are related by a unitary transformation U that does not factorize as $I_{A}⊗U_{1}⊗U_{2}$ relative to either decomposition. Physically, β and $\beta^{\prime }$ correspond to inequivalent identifications of the local degrees of freedom (for instance, different choices of qubits related by the action of an entangling unitary operator).

To define the meet $\alpha \wedge \beta^{\prime }$, we would need a TPS that is a common refinement of both. This would require that every tensor factor in $\alpha \wedge \beta^{\prime }$ appear as a factor in both a factor of α and a factor of $\beta^{\prime }$. Although both α and $\beta^{\prime }$ each have a proper refinement—$H_{A}⊗H_{B}⊗H_{C}$ and $H_{A}⊗H_{D}⊗H_{E}$, respectively—neither is a refinement of both. In the three-qubit case, although there are ways to decompose the entire space into TPSs with more factors than α and $\beta^{\prime }$, the subalgebras of local observables for α and $\beta^{\prime }$ do not commute pairwise; hence, there is no TPS whose factors simultaneously appear as factors in factors of both α and $\beta^{\prime }$. Consequently, meets do not generally exist in $T\left(H\right)$.

Note that a meet cannot be found even in cases where TPSs are only partially incompatible, for instance β and $\beta^{\prime }$ sharing $H_{A}$ as a common factor. Meets only exist within families of algebraically compatible partitions. As stated, this is not clearly the general case. Please note that this failure is not an artifact of low dimension, as one might be led to think if the case involved only two qubits (*B* and *C* in the example above). Even for a system as simple as three qubits, the space $T\left(H\right)$ already exhibits a purely algebraic obstruction. In higher-dimensional Hilbert spaces, and even more clearly in infinite-dimensional ones, incompatible TPSs abound.

On the other hand, the join $\alpha \vee \beta^{\prime }$ is the finest TPS that is coarser than both α and $\beta^{\prime }$. This requires that each factor of α and $\beta^{\prime }$ appear as a factor of some factor of the join. In this three-qubit example, given that α and $\beta^{\prime }$ are bipartite TPSs, there is no common coarsening TPS other than H itself, corresponding to the partition $\left\{U\right\}$. For more fine-grained TPSs that are only partially incompatible, such as the tripartite TPSs $H_{A}⊗H_{B}⊗H_{C}$ and $H_{A}⊗H_{D}⊗H_{E}$ above, a common coarsening finer than H can be found. In this case, the join is given by the tensor product of $H_{A}$ and the remaining four-dimensional factor. However, joins finer than the total system are not always available. For instance, if we take $H_{A}⊗H_{B}⊗H_{C}$ and mix the degrees of freedom of the three subsystems by means of an entangling unitary that does not factorize as $U_{1}⊗U_{2}⊗U_{3}$, we obtain a completely incompatible tripartite TPS: $H_{F}⊗H_{G}⊗H_{H}$. The join of these two TPSs cannot be anything other than H itself, since it is not possible to construct a bipartite TPS whose factors are themselves products of factors from both TPSs. Although this suffices to guarantee the existence of joins in $T\left(H\right)$ even for completely incompatible TPSs, it comes at the cost of a severe structural loss: intermediate elements are generally no longer connected to the top element through nontrivial common coarsenings.

The overall picture is as follows. As long as we restrict ourselves to families of algebraically compatible partitions, we can find locally lattice-like structures, each with its own meet operation and least element. However, one can construct pairwise incompatible decompositions—related, for instance, by generic entangling unitaries—for which no non-trivial common refinement or coarsening exists. This multiplicity undermines the uniqueness required for a single, global lattice-theoretic structure to be obtained. Although a top element can be clearly identified and a join operation is well-defined, there is neither a single least element nor a well-defined meet operation. Crucially, existing meets do not end in a unique least element, but in a plurality of incompatible atomic decompositions. Therefore, the poset $T\left(H\right)$ can no longer be globally considered a lattice. Rather, it resembles an ensemble of distinct lattice-like structures sharing $\left\{U\right\}$ as their common coarsening, branching into a plurality of algebraically incompatible atomic decompositions of that same whole.

The contrast with the classical case can now be stated more explicitly. In the extended framework based on classical mereology (Section 3.2), the global lattice-theoretic structure of the space of partitions plays a unifying role: any two ways of decomposing a whole (even when featuring in two different mereological hierarchies) can always be jointly compared, reconciled, and systematically related by taking their meet and join. This reflects a deep structural fact about classical partitions: they are organized by set-theoretic inclusion, and incompatibility between partitions is always resolvable by moving to finer or coarser divisions.

By contrast, the space of tensor product structures in Hilbert space has a structure that cannot be captured in lattice-theoretic terms. Incompatible TPSs need not admit a common refinement that preserves their subsystem structure, nor a common coarsening that subsumes them into a single higher-level decomposition, other than the total system as a whole. The obstruction is not merely epistemic or pragmatic, but structural and mathematically clearly expressible: different TPSs may correspond to different, mutually incompatible factorizations of the algebra of observables, and there is, in general, no TPS whose factors simultaneously stand in the parthood relations induced by both. The nature of this algebraic incompatibility between different decompositions has also been extensively studied in the algebraic quantum field theory (AQFT) tradition (e.g., Summers [23], Haag [24]). In this sense, incompatibility between quantum partitions is stronger than mere non-comparability in a partial order; it is a failure of lattice-theoretic closure. It is not only a matter that there are many mereological hierarchies, but they cannot be reconciled into a single, lattice-theoretic structure.

This reflects the big structural differences between quantum mechanics and classical mechanics. In classical mechanics, one may also perform canonical coordinate transformations on phase space, including those that introduce collective variables such as center-of-mass coordinates and relative distances, thereby mixing degrees of freedom. However, such transformations amount to mere reparameterizations of a single Cartesian product space $\Gamma_{A}\times \Gamma_{B}\times \Gamma_{C}$; they do not alter the identifiability of the original subsystems nor generate new degrees of freedom with independent physical significance. The Cartesian product structure remains fixed, and states are points (or probability distributions) for which there is no classical analog of superposition or entanglement. By contrast, in quantum mechanics, the tensor product structure $H_{A}⊗H_{B}⊗H_{C}$ permits, through global unitary transformations that do not factorize as $U_{A}⊗U_{B}⊗U_{C}$, a redefinition of the very notion of subsystem. One may construct new factorizations $H_{F}⊗H_{G}⊗H_{H}$, whose degrees of freedom are linear combinations of the original ones, such that the qubits before and after the transformation no longer correspond physically. This plasticity in the ways of partitioning the system, absent in classical mechanics, is a direct consequence of the structural features of Hilbert space.

The above reflections do not entail that the space of quantum partitions lacks a unifying structure. It simply means that the lattice-theoretic structure induced by classical mereology fails to capture it. The lack of a lattice-theoretic structure in the case of the space of quantum partitions cannot be taken by itself as an indication that quantum mereology should have non-extensional features. Nonetheless, it clearly points to the need for a new mereology for quantum mechanics—one that, when extended to the full space of quantum partitions, properly captures its unifying structure. Arguably, if we replace classical mereology with a new one to account for quantum parts, we would obtain a different picture when we move to the extended framework dealing with the whole space of partitions.

Although we still do not have a formal representation, in logical and mereological terms, of the aforementioned full space of quantum partitions—we only have access to it through Hilbert-space geometrical and algebraic features—we can infer some properties that it would have once fully developed. The properties of the full space of quantum partitions can function as a heuristic guide in the search for a new mereology for quantum mechanics, particularly one based on tensor product structures. In the following section, we show that the lack of lattice-theoretic structure may be related to the non-extensional behavior that a quantum mereology would exhibit when extended to the full space of partitions.

### 4.3. Global Failure of WSP in the Space of TPSs

In Section 4.2, we argued that the space $T\left(H\right)$ of the tensor product structures (TPSs) for a given Hilbert space does not form a single lattice structure under the refinement order. That structural difference from the classical partition lattice already signals a departure from classical mereology. However, to appreciate more concretely the non-classical nature that a TPS-grounded quantum mereology should have, we must examine whether some form of supplementation principle remains valid in $T\left(H\right)$.

In classical extensional mereology, the weak supplementation principle (WSP) plays a pivotal role. As recalled in Section 3.1, WSP states that if an entity has a proper part, it must have another part disjoint from the first. In the context of mereological partitions—ways of dividing a whole into parts—a natural analog of WSP can be formulated, provided we first clarify what it means for two partitions to be “disjoint.”

Recall from Section 3.2 that, in the extended framework built upon classical mereology, the set of partitions $\Pi \left(u\right)$ of a whole *u* forms a bounded lattice $L\left(u\right)$ under the refinement order $\underline{≺}$. Within this lattice, two partitions can be compared not only by granularity but also by whether they “overlap” in the sense of sharing non-trivial substructure. Intuitively, two partitions are disjoint if they carve up the whole in completely independent ways, with no common subdivision beyond the absolute finest partition (the atomic partition). Following the standard lattice-theoretic notion of disjointness, the idea is captured formally by using the meet operation of the lattice:

▪ Strong Disjointness of Partitions: Let ε,γ be two mereological partitions of a whole *u*. We say ε and γ are strongly disjoint, denoted $\varepsilon {⊥}_{S}\gamma$, if their meet equals the bottom element $\chi_{u}$ (the atomic partition):

(16) $$\varepsilon {⊥}_{S}\gamma \equiv \left(\varepsilon \wedge \gamma =\chi_{u}\right)$$

Since the meet ε∧γ is the coarsest partition that refines both ε and γ, having it equal to the atomic partition means that the only parts common to both decompositions are the atoms of *u*. In other words, the two partitions share no non-trivial mereological substructure; they are “orthogonal” decompositions of the whole. This definition respects the intuitive meaning of disjointness in a mereological partitions context: two ways of dividing the whole are disjoint if they do not agree on any composite part beyond the ultimate constituents.

With this strong notion of disjointness in hand, we can formulate a natural generalization of WSP that applies to partitions rather than to individual parts:

▪ Extended Weak Supplementation Principle (eWSP*_S_*):

$$\forall \varepsilon ,\phi \left(\varepsilon ≺\phi \to \exists \gamma \left(\gamma \underline{≺}\phi \wedge \left(\varepsilon {⊥}_{S}\gamma \right)\right)\right)$$

For any pair of partitions ε,ϕ, if ε is a proper refinement of ϕ (written ε≺ϕ), then there exists a partition γ such that

(1) $\gamma \underline{≺}\phi$ (i.e., γ is a refinement of ϕ);
(2) γ is strongly disjoint from ε: $\varepsilon {⊥}_{S}\gamma$.

The intuitive content of eWSP*_S_* mirrors that of the standard WSP. If a partition ε introduces strictly more structure than ϕ, then ϕ cannot be “exhausted” by ε alone: there must be some further way of refining ϕ that is independent of ε. In other words, a non-trivial refinement always leaves room for an alternative, disjoint refinement at the same level of coarse graining.

In the classical partition lattice, eWSP*_S_* is readily seen to hold. Indeed, if ε≺ϕ in $L\left(u\right)$, one can always construct a partition γ by taking, for each block of ϕ, a repartitioning that differs from the one induced by ε while still refining ϕ. The lattice structure guarantees the existence of such a γ, and one can always choose it so that its meet with ε is the atomic partition.

The situation is different for the space $T\left(H\right)$ of tensor product structures. As argued in Section 4.2, $T\left(H\right)$ is not globally a lattice. Although within families of compatible partitions, meets can be found, meets of arbitrary TPSs need not exist. This has direct consequences for the validity of eWSP*_S_*. Consider two distinct TPSs ε and ϕ of the same Hilbert space H, such that one is a proper refinement of the other—say ε≺ϕ—meaning every factor of ε is a factor of some factor of ϕ (i.e., ε arises from further factorizing some factors of ϕ). According to eWSP*_S_*, there should exist some TPS γ that also refines ϕ and is disjoint from ε. Disjointness in the strong sense defined above would require that the meet of ε and γ (understood as the greatest common refinement) equals the finest possible tensor decomposition. Although there is no TPS that plays the role of the atomic partition in an absolute sense, playing the role of the least element $\chi_{u}$ in a global lattice, certainly there is a fine decomposition refining all partitions within the family of compatible partitions to which ε and ϕ belong. In fact, there exists a partition γ with the same level of granularity as ε that belongs to the same family of compatible partitions but to a different mereological hierarchy within that family, such that the meet ε∧γ equals the least element relatively to that family. Hence, eWSP*_S_* remains valid in $T\left(H\right)$ only locally, relatively to a given family of compatible decompositions. In contrast, when we consider the full space of quantum decompositions, the disjointness condition ${⊥}_{S}$ cannot be satisfied in $T\left(H\right)$ in the strict sense: there is no absolute least element that would allow us to tell if two partitions are disjoint or not in an absolute sense.

As seen in Section 4.2, two algebraically incompatible TPSs are independent in the sense that they only share a trivial common coarsening, with no common refinement at all. If there is no common refinement, it is clear that they cannot share a common substructure. In fact, incompatible partitions in $T\left(H\right)$ are disjoint in the intuitive sense, but the definition of strong disjointness fails to capture this because they lack a meet. We can weaken the notion of disjointness to include cases where a common refinement cannot be found. The definition of disjointness now reads:

▪ Weak Disjointness of Partitions: Let ε,γ be two mereological partitions of a whole *u*. We say ε and γ are weakly disjoint, denoted $\varepsilon {⊥}_{W}\gamma$, if they have no meet or their meet admits no refinement:

(17) $$\varepsilon {⊥}_{W}\gamma \equiv \left(\varepsilon \wedge \gamma =\emptyset \right)\vee \neg \exists \chi \left(\chi \underline{≺}(\varepsilon \wedge \gamma )\right)$$

Since the meet ε∧γ should be the coarsest partition that refines both ε and γ, being disjoint in this weak sense means that there is no common refinement at all or that the only parts common to both decompositions are the atoms of *u* relative to a given family of compatible decompositions. In other words, the two partitions share no non-trivial mereological substructure.

Let us assess how the extended weak supplementation principle behaves comparatively under the weak and strong notions of disjointness. We shall denote the principle defined under weak disjointness by eWSP*_W_* to distinguish it from its counterpart defined under strong disjointness (eWSP*_S_*). Suppose α,β and $\beta^{\prime }$ are as above. Consider first β as a proper refinement of $\left\{U\right\}$. To satisfy the extended weak supplementation principle, we would need a TPS γ refining $\left\{U\right\}$ that is disjoint of β. Since $\alpha \wedge \beta =H_{A}⊗H_{B}⊗H_{C}$ admits no refinement, then α=γ, both under eWSP*_W_* and, locally, under eWSP*_S_*. Now consider $\beta^{\prime }$ as a proper refinement of $\left\{U\right\}$. We would need a TPS γ refining $\left\{U\right\}$ that is disjoint of $\beta^{\prime }$. Since $\alpha \wedge \beta^{\prime }=\emptyset$, then α=γ, but only under eWSP*_W_*. Under eWSP*_S_*, a possible γ can only be found within the same family of compatible partitions, for instance $\left(H_{A}⊗H_{D}\right)⊗H_{E}$. Note that the meet of this partition with $\beta^{\prime }$, that is $H_{A}⊗H_{D}⊗H_{E}$, admits no refinement.

In general, the non-lattice character of $T\left(H\right)$ entails that such a γ may simply not exist under eWSP*_S_*, outside a given family of compatible partitions. The subalgebras of observables associated with a given partition and any candidate γ outside its own family of compatible partitions will not commute, meaning there is no consistent way to embed both decompositions into a single refinement. The obstruction is algebraic and fundamental: different TPSs may correspond to different ways of splitting the total algebra of observables into commuting subalgebras, and these splittings can be mutually incompatible. As a result, for a given ε≺ϕ, there may be no disjoint TPS $\gamma \underline{≺}\phi$ outside its own family of compatible partitions. Hence, eWSP*_S_* is only locally valid, relative to a certain lattice-like structured family of compatible partitions with its own finest refinement, while globally failing in $T\left(H\right)$.

Since weak disjointness (Equation (17)) does not differentiate between disjoint algebraically compatible and incompatible partitions, eWSP*_W_* remains globally valid in $T\left(H\right)$. Moreover, under such a weaker notion of disjointness, even an extended version of the strong supplementation principle would turn out to be globally valid in $T\left(H\right)$, perhaps rather trivially. On the other hand, it is true that the strong notion of disjointness first proposed (Equation (16)) is seemingly rather lattice-theoretic dependent, requiring that a meet exists that happens to be the least element. From this perspective, it is not a surprise that extended supplementation principles fail in spaces lacking a lattice-theoretic structure and hold in those exhibiting precisely that structure. The adoption of a weak notion of disjointness would lack that compromise, but at the cost of blurring the structural difference between the space of classical partitions and the space of quantum partitions. It looks like an ad hoc movement to keep the space of TPSs classical even when it is not a single lattice. Another problem that the weak notion of disjointness has is that it misses cases where two TPSs are only partially incompatible, as our β and $\beta^{\prime }$ above. Since they have no common refinement, they would be taken as disjoint partitions, even when they share a common tensor factor. For these reasons, we prefer to adopt the strong notion of disjointness in order to capture the two different ways in which extended supplementation principles can be valid in a space of partitions: globally and locally. With the proposed notions at hand, eWSP*_S_* has full global validity in the space of classical partitions while being at most locally valid in the space of quantum partitions.

Both the local validity and global failure of eWSP*_S_* in the space of tensor product structures have interesting implications for the status of supplementation principles in quantum mereology. Recall that in classical extensional mereology, the strong supplementation principle (SSP) entails the weak supplementation principle. If eWSP*_S_*—the natural partition-level analog of WSP—globally fails, then an analogous strong supplementation principle for partitions must globally fail as well. More concretely, if there exist refinements of a whole that cannot be “supplemented” by disjoint alternatives in a strong, absolute sense, then the space of mereological partitions in $T\left(H\right)$ does not support the kind of extensional behavior built into the classical lattice. In other words, the failure of eWSP*_S_* indicates that the space of admissible quantum decompositions is too rich and too structured to satisfy the global supplementation conditions that underpin extensional mereology.

But, on the other hand, it seems reasonable that a classical-like, extensional mereology can be recovered when we restrict ourselves to a fixed family of compatible partitions and consider only the subsystem decompositions it affords. That would be a local, internal form of extensional mereology (as Calosi and Tarozzi suggest). The situation would be analogous to that of quantum logic: although it is globally non-extensional (the algebra of projections in the full Hilbert space is non-Boolean), classical extensional (Boolean) logic is recovered when we restrict ourselves to sets of commuting projections.

The resulting picture is as follows. Once a quantum mereology is in place, one expects that, when extended to the full space $T\left(H\right)$ of all possible tensor product structures, eWSP*_S_* will fail globally (and hence so will a corresponding partition-level SSP), thereby revealing a certain non-extensional character of extended quantum mereology. Quantum mereology, when grounded on TPSs, should not simply mirror classical mereology with a lattice-theoretic structure binding all possible mereological hierarchies; it should, when extended, give rise to a unified framework encompassing a plurality of mutually algebraically incompatible families of partitions that cannot be jointly regimented into a single supplemented lattice. This “built-in” pluralism undermines the extensional tenet that the identity of the whole is uniquely determined by the identity of its parts because what counts as a “part” is relative to a decomposition in a much stronger sense: in principle, there is no satisfactory absolute notion of disjointness, only one relative to a choice of a family of compatible TPSs.

This sharp divergence from the classical partition lattice shows that extensionality cannot be globally maintained once the full space of admissible quantum decompositions is taken into account. While each fixed TPS may support an internal, extensional mereology of subsystems, the total mereological space generated by all possible TPSs does not. Classical mereology gives rise to a framework in which all legitimate partitions of a whole fit together into a single lattice. In contrast, TPS-grounded quantum mereology, when extended, should capture a structure that is strictly richer than its classical counterpart. Its non-lattice character provides a sense in which extended quantum mereology, even when grounded on tensor factors, exhibits genuinely non-classical features. Extensional conclusions drawn by Calosi and Tarozzi rely on an implicit restriction to a single, privileged family of compatible decompositions rather than on the full structure permitted by quantum theory.

In summary, the violation of the extended weak supplementation principle in the space of tensor product structures, under strong disjointness, provides a further formal criterion distinguishing quantum mereology from its classical counterpart. It reinforces the conclusion of Section 4.2: the extended mereological framework for quantum partitions is not extensional in the classical sense, and its proper formalization must account for the non-lattice, supplementation-violating structure of the space of possible decompositions.

## 5. Conclusions

In this paper, two broad approaches to quantum mereology have been surveyed: one based on the lattice of Hilbert subspaces (or convex sets of states) and one based on tensor factors. While the former offers a rich mereological framework for reasoning about quantum properties, which is naturally aligned with quantum logic, the latter aligns more closely with operational physical practice, in which composition is modeled via tensor products and subsystems correspond to tensor factors. Our focus has been on this second approach, which we regard as a direct formalization of the part–whole relations actually employed in standard physical practice.

We have shown, however, that even within this TPS-grounded framework, quantum mereology should depart from classical extensional mereology in certain ways. The central conceptual move has been to extend mereology to treat spaces of mereological partitions. In this way, the notion of part is extended to obtain a notion of mereological partition; the relation of parthood is extended to obtain a relation of refinement; and the notion of disjointness between parts is extended to obtain a notion of disjointness between partitions. Thus, when treating tensor factorizations as mereological partitions of a quantum whole, we found that, while each family of mutually algebraically compatible partitions may internally satisfy classical mereological axioms—as argued by Calosi and Tarozzi [13]—the space of all possible tensor product structures for a given Hilbert space lacks the lattice-theoretic organization characteristic of classical partitions. For this reason, we proposed that the development of a quantum mereology must move beyond classical extensional frameworks and take seriously the structural particularities imposed by the tensor product structures (TPSs) of Hilbert space to the space of mereological partitions.

Specifically, we argued in Section 4.2 that the set $T\left(H\right)$ of TPSs does not globally form a lattice, meets are generally not well-defined, and different decompositions can be incompatible in a strong sense. In Section 4.3, we further developed this analysis to show that an extended weak supplementation principle (eWSP*_S_*), formulated for partitions under a strong notion of disjointness between partitions, fails globally in the space of TPSs. Since supplementation principles are necessary for extensionality, this failure entails that a TPS-grounded quantum mereology, when extended to the full space of partitions, cannot be globally extensional in the classical sense.

These results challenge the view that quantum composition can be modeled within already extant classical extensional mereology and instead support the project of developing a new mereological framework for quantum composition that accommodates the non-extensional character of the space of quantum partitions, understood globally. The non-extensionality we identify is not merely a consequence of quantum holism or entanglement, but stems from the strong relativity of quantum decomposition: what counts as a subsystem—and thus as a part—crucially depends on the choice of a family of algebraically compatible tensor product structures, with no single family of decompositions being privileged by the Hilbert space alone and no single atomic decomposition. This has no analog in the classical partitions lattice, where, although there is a plurality of different mereological hierarchies, they are all connected via lattice-theoretic operations, with a unique least element playing the role of the atomic partition.

A full-fledged quantum mereology extended to the space of all quantum partitions must therefore accommodate this decompositional relativity, perhaps by indexing parthood relations to specific TPSs, or by adopting a metatheoretical framework other than standard set theory. Rather than seeking to impose classical parthood relations onto quantum systems, we should build a brand new mereology, satisfying two key desiderata: first, to capture the non-extensional, non-lattice-theoretic structure of quantum partitions, and second, to recover extensionality and classical-like behavior when restricted to a single family of compatible partitions.

Looking forward, we suggest that the property-based mereology of Holik and Jorge [7] and the subsystem-based mereology grounded in TPSs should not be seen as rivals, but as complementary perspectives. The former enriches our understanding of quantum properties and their logical structure; the latter formalizes the compositional relations used in physical modeling. Both face distinct formal challenges—the convex-state approach must extend quantum logic, while the TPS-based approach must contend with a non-lattice space of decomposition, and both illuminate different aspects of what it means for a quantum system to have parts.

## Data Availability

No new data were generated as part of this study.
